# An investigation of the construct validity of the ICECAP-A capability measure

**DOI:** 10.1007/s11136-012-0293-5

**Published:** 2012-10-20

**Authors:** Hareth Al-Janabi, Tim J. Peters, John Brazier, Stirling Bryan, Terry N. Flynn, Sam Clemens, Alison Moody, Joanna Coast

**Affiliations:** 1 Health Economics Unit, School of Health and Population Sciences, Public Health Building, University of Birmingham, Edgbaston, Birmingham, B15 2TT UK; 2 School of Clinical Sciences, University of Bristol, Bristol, UK; 3 School of Health and Related Research, University of Sheffield, Sheffield, UK; 4 School of Population and Public Health, University of British Columbia, Vancouver, BC Canada; 5 Centre for the Study of Choice, University of Technology Sydney, Sydney, Australia; 6 National Centre for Social Research, London, UK; 7 Institute of Epidemiology and Health Care, University College London, London, UK

**Keywords:** Capability approach, Health economics, Outcomes, Psychometrics, Quality of life, Wellbeing

## Abstract

**Purpose:**

To investigate the construct validity of the ICECAP-A capability wellbeing measure.

**Methods:**

A face-to-face interview-administered survey was conducted with 418 members of the UK general population, randomly sampled from the Postcode Address File. Pre-specified hypotheses were developed about the expected associations between individuals’ ICECAP-A responses and their socio-economic circumstances, health and freedom. The hypotheses were investigated using statistical tests of association.

**Results:**

The ICECAP-A responses and scores reflected differences across different health and socioeconomic groups as anticipated, but did not distinguish individuals by the level of local deprivation. Mean ICECAP-A scores reflected individuals’ perceived freedom slightly more closely than did measures of health and happiness.

**Conclusion:**

This study suggests that the ICECAP-A measure can identify expected differences in capability wellbeing in a general population sample. Further work could establish whether self-reported capabilities exhibit desirable validity and acceptability in sub-groups of the population such as patients, social care recipients and informal carers.

## Introduction

The capability approach advocates assessing wellbeing in terms of individuals’ ‘functionings’ and ‘capabilities’. Functionings are the things that an individual ‘is’ or does’ and can be broadly defined, potentially ranging from elementary aspects of their life such as ‘being adequately nourished’ and ‘having good health’ to more complex aspects such as ‘achieving self-respect’ or ‘being socially integrated’[1]. Capabilities represent an individual’s freedom to carry out these functionings, whether or not the individual chooses to do so. Interest in using the capability approach in the health field has grown in recent years, with authors proposing it as a framework for conceptualising health [2, 3] and disability [4, 5], measuring intervention outcomes [6, 7] and assisting in decisions about healthcare resource allocation [8, 9].

Despite much interest in the capability approach, few practical measures of capability have been developed. Indeed, some authors question the degree to which capability measurement is possible with such a rich array of potential functionings and disagreement on the functionings that constitute a ‘good life’ [10]. Nevertheless, recent work has been conducted to develop measures of capability as a way of operationalising the capability approach. One stream of work has sought to develop capability ‘indicators’ using existing survey questions [11], relating to Martha Nussbaum’s list of central human capabilities [12]. Another approach to operationalising the capability approach is in using interviews with the public to generate a set of core capabilities, which can then be assessed using short, self-completion questionnaires, such as the ICECAP measures [13, 14].

An important challenge in the development of all measures is the assessment of validity. If it can be demonstrated that measures reflect what they purport to, then greater confidence can be placed in results generated. Capability is a particularly challenging trait for which to develop valid measures. First, the scope of capability measures is potentially quite broad. In principle, one could demand that any capability measure needs to demonstrate responsiveness to a huge array of factors before it can be considered valid. Second, capability measurement implies the quantification of something that is unobservable [15]: the freedom or opportunities available to an individual. It requires an *ex ante* assessment, focusing on what an individual has the freedom/potential to do, rather than an *ex post* assessment of what they do in fact do.

Some validation work has been conducted with the ICECAP-O capability measure, developed for older populations [16, 17], and this has focused on examining factors anticipated to be associated with functioning *per se* rather than freedom to function. The aim in the study described here was to investigate the construct validity of a new instrument, recently developed to measure capability wellbeing for the general adult population: the ICECAP-A (ICEpop CAPability measure for Adults) [13]. The ICECAP-A (reproduced in ‘Appendix’) has been designed to capture capability to function across five attributes of life: ‘stability’, ‘attachment’, ‘autonomy’, ‘achievement’ and ‘enjoyment’. Individuals are asked to select the level of capability (from four options) that corresponds with their situation across each of the five attributes. This paper reports a series of investigations of the construct validity of the ICECAP-A descriptive system and index scores, focussing on associations between reported capability and individuals’ socio-economic circumstances and health status. The paper also reports an investigation of the, more challenging, issue of whether the ICECAP-A measure appears to be reflecting individual perceptions of their freedom in life.

## Methods

The data for this study come from a face-to-face interview-administered survey, conducted by the National Centre for Social Research (NatCen) in the UK. In this survey, the ICECAP-A measure and a range of contextual questions were asked. The survey questions covered: (1) socio-demographics, (2) measures of material wellbeing (income, home ownership), (3) major life events (bereavement, relationship break-up, etc.) in the last 6 months, (4) happiness and religiosity, (5) health, (6) use of healthcare, (7) perceptions of freedom.

Respondents were randomly selected for the survey from the Postcode Address File (PAF) in Great Britain using a two-stage stratified random sample design (the PAF was stratified on the basis of geographic area and socio-economic deprivation). The sample of 802 addresses was selected with the aim of obtaining at least 400 responses for the valuation survey, based on prior experiences of response rates from NatCen surveys. At each selected address, one adult was randomly selected to take part in the survey. Each address was sent a postal invitation to participate, which the designated interviewer followed up in person. Up to nine attempts were made to make contact and confirm whether the selected individual wished to participate. The survey was administered by NatCen interviewers using computer-assisted personal interviewing (CAPI) software. Interviewers received specific training on the content and purpose of the survey and procedures to use when making contact, gaining consent and interviewing participants. The study protocol was approved by the University of Birmingham’s Life and Health Sciences Ethical Review Committee (ERN_08-93).

Good practice in validating measurement tools demands that hypotheses are developed in advance regarding the (expected) relationship between the trait (capability) and relevant contextual factors [18]. In this study, a network of constructs identifying factors likely to be associated with each of the five capabilities was developed. These hypotheses drew partly on the qualitative research to develop the ICECAP-A measure [13]. This qualitative work set out to go beyond identifying influences on wellbeing, such as work or income, to examine why these factors were important in individuals’ lives. As a result, the qualitative data provide a rich source of information to identify hypotheses about the anticipated relationship between the influences on wellbeing that individuals tended to discuss in interview (for instance, work) and the ultimate capability that this helped to facilitate (such as *achievement* or *stability*). Hypotheses also drew on research relating to the validity of related quality of life measures [16, 17, 19, 20] and on the general wellbeing literature [21].

Associations between the five capability responses and the background variables were investigated using chi-squared tests for categorical variables and one-way analysis of variance for continuous variables. For categorical variables, where a number of cell counts were < 5, exact tests were used when computationally feasible; where they were not possible, variables were re-coded to increase cell counts. Alongside the direction of the relationship, the statistical strength of the evidence for each relationship was noted using significance levels of 5 and 1 %. All analyses were undertaken using Stata version 10.

### Investigation 1: Do measured capabilities reflect socio-economic circumstances?

Drawing on Sen’s conceptual framework for the creation of capabilities, it is clear that capability can be limited by poor socio-economic circumstances and enhanced by good circumstances [22]. For investigation 1, a table was drawn up showing the expected association between various indicators of socio-economic status and response to each of the ICECAP-A capabilities. All members of the research team contributed to this table. The section below details the conceptual capability in bold, the lay terms in the measurement instrument (also reproduced in ‘Appendix’) in italics and the anticipated associations with this capability.

- A capability for **stability** (*able to feel settled and secure*) relates to the absence of stress and dramatic changes in life and an ability to assign meaning to life. It was therefore anticipated that recent major negative life events (e.g. bereavement, relationship break-up, financial problems and serious ill health) were likely to be associated with reduced capability in this area. It was also predicted that being employed, being in a permanent relationship, having a good income and living in a low crime area were all likely to be associated with higher capability in this domain.
- A capability for **attachment** (*able to have love, friendship and support*) relates to the ability to interact with others and have high quality relationships. It was therefore anticipated that capability would be lower on this domain for individuals who reported recent relationship problems, separation or bereavement and higher for individuals who had a partner.
- A capability for **autonomy** (*able to be independent)* relates to being able to look after oneself, make one’s own decisions, and secure privacy and identity. It was anticipated that autonomy would be lower for those who were in relationships but higher for those with more education, those who were employed, those with higher income and home owners.
- A capability for **achievement** (*able to achieve and progress)* reflects individuals’ abilities to move forward in their life and attain their goals. This attribute also reflects perceptions of satisfaction and recognition. It was therefore anticipated that capability for achievement would be higher for individuals in employment, with more education, with higher incomes (and no recent financial worries) and those who had no recent break-up with a partner.
- A capability for **enjoyment** (*able to have enjoyment and pleasure)* reflects opportunities for the ‘quiet pleasures’ in life, such as enjoying nature, as well as things that are perceived to be ‘fun’ or ‘exciting’. As such, it was anticipated that the capability for enjoyment would be lower for individuals who reported negative recent life events, suffered unemployment or lived in an area with high crime rates, and a capability for enjoyment would be higher amongst individuals in relationships, those with higher incomes and those who reported high happiness levels.

### Investigation 2: Do measured capabilities reflect variations in health status?

The ICECAP capability measures were developed with an initial aim of measuring the effectiveness of health and social care interventions. The degree to which variations in health and health care usage are reflected in individuals’ capabilities is therefore of crucial interest and importance. The capability literature is fairly clear that poor health and disability plays an important role in limiting human capability [23]. Based on the qualitative work to develop the measurement tool and evidence from the ICECAP-O instrument [14, 17, 24, 25], it was anticipated that impairments to physical health would reduce capability for *stability, autonomy, achievement* and *enjoyment*, while impairments in mental health would additionally limit all five capabilities (including *attachment*). It was also anticipated that proxy measures of poor physical health—such as presence of a long-standing illness, receipt of hospital care and unpaid (informal) care—would be associated with impairments across *stability, autonomy, achievement* and *enjoyment*. Analysis to investigate these hypotheses proceeded in the same way as in ‘Investigation 1’.

### Investigation 3: Do measured capabilities reflect individual perceptions of freedom?

The investigations outlined in ‘Investigation 1’ and ‘Investigation 2’ would be relevant whether a measurement tool focuses on functioning or capability. Since this study focuses on the measurement of capability, we investigated the degree to which responses to the measure reflected an individual’s perceptions of their freedom in life. This was achieved by examining the association between responses to the capability measure and the three statements below about individuals’ perceived freedom.

- Life is full of opportunities (*often/sometimes/not often/never*)
- What happens to me is out of my control (*often/sometimes/not often/never*)
- I can do the things in life I want to do (*often/sometimes/not often/never*)

These questions were not intended to represent a ‘gold standard’ assessment of freedom. However, at the very minimum, it was anticipated that individuals who indicated higher levels of freedom would indicate higher levels of capability. This was investigated by calculating the magnitude and strength of evidence of the associations between responses to the freedom questions and an individuals’ ICECAP-A index score [26]. Since one may expect individuals reporting greater freedom and control to report higher levels of wellbeing (however measured), a more exacting test of a capability measure is whether it correlates *more closely* with measures of freedom than alternative outcome measures that focus on functioning. To examine this issue, the pair-wise (Pearson) correlation coefficients between the freedom questions and the ICECAP-A measure were compared to those between the freedom questions and two prominent techniques for measuring outcomes in health economics: the EQ-5D health measure [27] and a global subjective ‘happiness’ question. The EQ-5D is a self-report measure of generic health status, focussing on five attributes of health: mobility, self-care, usual activities, pain/discomfort and anxiety/depression. The happiness question required respondents to select one of three statements that referred to their situation (‘Taking all things together, how would you say you are these days—would you say you’re very happy, fairly happy, or not too happy these days?’). It was hypothesised that the correlations between the freedom variables and ICECAP-A (capability) measure would be stronger than those between the freedom variables and the EQ-5D and happiness measures (which could both be conceived more as measures of functioning [22]).

### Do different capabilities measure different things?

Since the capability approach generally (and the ICECAP-A instrument specifically) is multidimensional, we investigated whether different capabilities were tapping into different constructs. We hypothesised that certain capabilities should be more highly associated with specific characteristics than other capabilities. Therefore, while both *autonomy* and *achievement* might correlate with both self-care problems (on the EQ-5D) and educational level, we hypothesised that the stronger relationships would be between self-care and *autonomy* and between education and *achievement*. Drawing on the key influences on each attribute noted in the qualitative research (and based on variables that were available), one background variable was selected for each of the five ICECAP-A capabilities and hypothesised to correlate more highly with a selected capability than the other four. These hypotheses were examined through calculating correlation coefficients between the background variables and the capabilities.

## Results

Survey interviews were completed between March and June 2010. From the 802 addresses selected, 422 (52 %) individuals responded, and of these 418 (99 %) produced complete interviews. Descriptive statistics for the sample are provided in Table 1. All 418 individuals who reached the end of the survey fully completed the ICECAP-A capability measure (Table 2). The modal response of the ICECAP-A was the top or second level of capability across each of the five attributes. Nevertheless, many individuals indicated that their capability was highly limited (little capability or no capability) on each of the five attributes. This ranged from 37 individuals (8 %) on *attachment* to 120 individuals (28 %) on *achievement*.

**Table 1:** Sample characteristics (*n* = 418)

Characteristics	Category	Frequency
Socio-demographics		
Age (*n* = 416) [Mean: 51.7, SD 18.2]	18–29	55 (13 %)
	30–44	106 (25 %)
	45–64	133 (32 %)
	65+	122 (29 %)
Sex (*n* = 418)	Female	259 (62 %)
	Male	159 (38 %)
Education (*n* = 418)	No certificated qualifications	134 (32 %)
	With certificated qualifications	284 (68 %)
Marital status (*n* = 417)	Married (or de facto married)	191 (46 %)
	Cohabiting	36 (9 %)
	Single	72 (17 %)
	Widowed	49 (12 %)
	Divorced	49 (12 %)
	Separated	15 (4 %)
	Civil partnership	5 (1 %)
Home ownership (*n* = 415)	Own outright	141 (34 %)
	Mortgage/loan	123 (30 %)
	Part own/part rent	10 (2 %)
	Rent	132 (32 %)
	Rent-free	9 (2 %)
Annual household income (*n* = 357)	<£10,000	78 (22 %)
	£10-19,999	102 (29 %)
	£20-£29,999	53 (15 %)
	£30-£49,999	67 (19 %)
	£50-£74,999	37 (10 %)
	£75,000+	20 (6 %)
Employment (*n* = 414)	In paid employment	201 (49 %)
	Not in paid employment (retired, homemaker, long term sick etc.)	213 (51 %)
Other	1	190 (45 %)
EQ-5D (*n* = 418)	0.75–1	110 (26 %)
	0.5–0.75	84 (20 %)
	<0.5	34 (8 %)
Happiness (*n* = 418)	Very happy	116 (28 %)
	Fairly happy	216 (52 %)
	Not too happy	36 (9 %)

**Table 2:** Response to ICECAP-A questionnaire (*n* = 418)

Attribute	Frequency (%)
*Stability*	
I am able to feel settled and secure in all areas of my life	120 (29 %)
I am able to feel settled and secure in many areas of my life	215 (51 %)
I am able to feel settled and secure in a few areas of my life	71 (17 %)
I am unable to feel settled and secure in any areas of my life	12 (3 %)
*Attachment*	
I can have a lot of love, friendship and support	252 (60 %)
I can have quite a lot of love, friendship and support	129 (31 %)
I can have a little love, friendship and support	31 (7 %)
I cannot have any love, friendship and support	6 (1 %)
*Autonomy*	
I am able to be completely independent	191 (47 %)
I am able to be independent in many things	171 (41 %)
I am able to be independent in a few things	47 (11 %)
I am unable to be at all independent	5 (1 %)
*Achievement*	
I can achieve and progress in all aspects of my life	75 (18 %)
I can achieve and progress in many aspects of my life	223 (53 %)
I can achieve and progress in a few aspects of my life	110 (26 %)
I cannot achieve and progress in any aspects of my life	10 (2 %)
*Enjoyment*	
I can have a lot of enjoyment and pleasure	154 (37 %)
I can have quite a lot of enjoyment and pleasure	193 (46 %)
I can have a little enjoyment and pleasure	61 (15 %)
I cannot have any enjoyment and pleasure	10 (2 %)

### Investigation 1: Do measured capabilities reflect socio-economic circumstances?

Table 3 shows the associations between contextual characteristics of individuals’ lives and their capabilities. Associations that were hypothesised a priori are highlighted in italics. The remaining associations are listed for completeness. Of the 55 hypothesised associations: twenty-nine (53 %) were in the expected direction and had *p* < 0.01; five (9 %) were in the expected direction and had *p* < 0.05 (but > 0.01); 21(38 %) had *p* ≥ 0.05; none were in an unexpected direction and had *p* < 0.05.

**Table 3:** Univariable associations between ICECAP-A attributes and individuals’ characteristics

Contextual characteristics	Stability	Attachment	Autonomy	Achievement	Enjoyment
*Socio*-*demographics*					
Age	0.72	0.77	*0.16*	*0.089*	0.71
Sex	0.91	0.68	0.48	0.94	0.61
Employment	0.003**	0.028*	<0.001**	<0.001**	<0.001**
Education/qualifications	0.019*	0.41	0.001**	0.009**	0.076
Relationship status	<0.001**	<0.001**	*0.31*	0.003**	0.013*
Home ownership	0.003**	0.044*	<0.001**	<0.001**	0.003
Income	<0.001**	0.002**	0.036*	<0.001**	<0.001**
IMD barriers to housing	*0.73*	0.94	*0.87*	*0.99*	0.28
IMD local crime	*0.47*	0.14	0.089	0.93	0.14
IMD housing and environment quality	0.23	0.19	0.16	0.79	*0.058*
*Major life events (experienced in last 6* *months)*					
Bereavement	*0.20*	*0.20*	0.038	0.52	*0.61*
Break-up	<0.001**	0.008**	0.020	0.001**	0.005**
Household job loss	*0.099*	0.18	0.028	0.93	0.036
Job change	0.008**	0.53	0.059	0.59	0.76
Financial worries	<0.001**	0.079	0.15	0.009**	0.002**
Moved house	0.029*	*0.061*	0.87	0.13	0.35
Problems with relatives	0.001**	0.011*	0.66	0.034	0.033*
Problems with children	*0.095*	*0.12*	0.74	0.32	0.006**
Problems at work	0.005**	*0.058*	0.5	0.22	*0.055*
Problems with neighbours	0.002**	0.001**	0.95	0.23	*0.14*
Serious accident	*0.43*	0.49	1.00	0.034	0.84
Serious illness	0.001**	0.188	0.001**	0.010**	*0.19*
*Attitudes*					
Happiness	<0.001**	<0.001**	<0.001**	<0.001**	<0.001**
Religiosity	*0.60*	0.37	0.033*	0.60	0.10

Italic cells are those where an association was hypothesised a priori

*IMD* index of multiple deprivation

* significant (in the expected direction) at the 5 % level

** significant (in the expected direction) at the 1 % level

Broadly speaking, there were statistically significant associations, where anticipated, between measured capability and employment, education, relationship status, home ownership, income, major life events (with some exceptions) and happiness.

However, associations were not found, although they were anticipated, between capability and indicators of local deprivation, religiosity or having a recent bereavement, household job loss or accident. No relationship was hypothesised between sex (gender) and capability, and none was found.

### Investigation 2: Do measured capabilities reflect variations in health status?

Table 4 shows the expected association between various indicators of health (and health care use) and each of the ICECAP-A capabilities. As for ‘Do measured capabilities reflect socio-economic circumstances?’, associations that were hypothesised are highlighted in italics, with remaining associations listed for completeness. To summarise, for the 42 hypothesised associations: thirty-two (76 %) were in the expected direction and had *p* < 0.01; one (2 %) was in the expected direction and had *p* < 0.05 (but >0.01); eight (19 %) had *p* ≥ 0.05; one (2 %) was in an unexpected direction and had *p* < 0.01.

**Table 4:** Univariable associations between ICECAP-A attributes and health variables

Health variable	Stability	Attachment	Autonomy	Achievement	Enjoyment
*EQ-5D*					
EQ-5D index score	<0.001**	0.34	<0.001**	<0.001**	<0.001**
Mobility	<*0.001††*	0.10	<0.001**	<0.001**	0.003**
Self-care	0.005**	0.002	<0.001**	0.001**	0.002**
Usual activities	<0.001**	0.002	<0.001**	<0.001**	<0.001**
Pain	<0.001**	0.007	<0.001**	<0.001**	<0.001**
Anxiety/depression	<0.001**	<0.001**	<0.001	<0.001**	<0.001**
*Health-related variables*					
Longstanding illness	0.001**	0.42	<0.001**	<0.001**	<0.001**
Inpatient appointment in last year	*0.15*	0.84	*0.096*	*0.55*	*0.65*
Outpatient appointment in last year	*0.8*	0.91	*0.066*	0.003**	0.008**
Receive (formal/informal) care	0.023*	0.24	<0.001**	<0.001**	<0.001**
Provide informal care	0.50	*0.94*	*0.62*	0.29	0.078

Italic cells are those where an association was hypothesised a priori

* significant (in the expected direction) at the 5 % level

** significant (in the expected direction) at the 1 % level

†† significant (in the unexpected direction) at the 1 % level

The results indicate strong evidence for all but one of the hypothesised associations between the five capabilities and the EQ-5D attributes (and index score), in the anticipated direction. As hypothesised, *stability*, *autonomy*, *achievement* and *enjoyment* were associated with the four physical health attributes of the EQ-5D. *Attachment* (along with the other four capabilities) was associated with the answers to the mental health question about anxiety and depression. There was also evidence for hypothesised associations between the capabilities and the presence of a long-standing illness and receipt of care. Although associations between capabilities and inpatient/outpatient appointments and the provision of informal care were hypothesised, there was no evidence for them in this data set.

### Investigation 3: Do measured capabilities reflect individual perceptions of freedom?

Table 5 shows the mean ICECAP-A index score (with 0 indicating no capability on any attributes and 1 indicating full capability on all attributes) for individuals responding to the three questions about their freedom. Across each freedom question, higher levels of reported freedom are associated with higher capability scores. The effect is more pronounced when individuals are differentiated by their perceptions of their opportunities and ability to do what they want to do life. Table 6 confirms the strong statistical evidence of an association between individuals’ capability in general, and their perceptions of freedom. The table also indicates that health (as measured by the EQ-5D) and happiness are associated with perceptions of freedom. As hypothesised, freedom is slightly more closely correlated with the capability measure than the functioning measures. Table 7 shows the effect sizes for these differences in correlation. The only comparison demonstrating evidence of a difference is that between the capability and ‘doing things that I want’ correlation and the happiness and ‘doing things that I want’ correlation. Nevertheless, for four of the other five pairwise comparisons of correlation coefficients, there is a ‘small’ [28] difference (effect size of approximately 0.1) in favour of the capability measure in the correlation coefficients.

**Table 5:** Capability score by level of freedom

Variable	Mean (SD)
	ICECAP-A score
*Life is full of opportunities* (‘Opportunities’)	
Often (*n* = 144)	0.89 (0.13)
Sometimes (*n* = 194)	0.84 (0.12)
Not often (*n* = 65)	0.74 (0.17)
Never (*n* = 14)	0.54 (0.30)
*What happens to me is out of my control* (‘Control’)	
Often (*n* = 69)	0.73 (0.21)
Sometimes (*n* = 187)	0.83 (0.16)
Not often (*n* = 120)	0.88 (0.10)
Never (*n* = 41)	0.89 (0.11)
*I can do the things in life I want to do* (‘Do things I want’)	
Often (*n* = 195)	0.90 (0.10)
Sometimes (*n* = 170)	0.82 (0.13)
Not often (*n* = 46)	0.66 (0.10)
Never (*n* = 7)	0.49 (0.30)

**Table 6:** Correlations between ‘freedom’ variables and measures of capability and functioning (*n* = 416)

Variable	ICECAP-A	EQ-5D	Happiness
Opportunities	0.44**	0.34**	0.34**
Control	0.32**	0.31**	0.23**
Do things I want	0.54**	0.44**	0.30**

ICECAP-A and EQ-5D are scored using the Flynn et al. (2012) and Dolan et al. (1996) tariffs respectively

** significant at the 1 % level

**Table 7:** Effect sizes for differences in correlation with freedom questions between capability and functioning measures

Variable	ICECAP-A compared to	
	EQ-5D	Happiness
Opportunities	0.11	0.11
Control	0.009	0.10
Do things I want	0.13	0.29**

** significant at the 1 % level

Table 8 reports the correlation coefficients between the responses to the freedom questions and the five capability questions individually (all have *p* < 0.05 at least). From the table, it appears that perceptions of freedom are most strongly associated with capabilities for *achievement* and *enjoyment* and least strongly associated with the capability for *attachment*.

**Table 8:** Correlations between ‘freedom’ variables and ICECAP-A attributes (*n* = 416)

Variable	Stability	Attachment	Autonomy	Achievement	Enjoyment
Opportunities	0.23**	0.29**	0.20**	0.42**	0.43**
Control	0.27**	0.12*	0.22**	0.29**	0.25**
Do things I want	0.32**	0.29**	0.44**	0.46**	0.45**

** significant at the 1 % level

* significant at the 5 % level

### Do different capabilities measure different things?

Table 9 shows the pairwise correlations between the five selected background variables and the five ICECAP-A capabilities. The hypothesised strongest correlations (by row) are in the cells on the diagonal from top left to bottom right. In all cases, these correlations are in the expected direction and have *p* < 0.01. For three of the five capabilities (*stability, autonomy* and *achievement*), the selected background variable correlates more closely with that capability than the other four. In two cases (attachment and enjoyment), the correlation with the identified background variable is the second strongest correlation in the row (in both cases after the correlation of the background variable and *stability*).

**Table 9:** Correlations between ICECAP-A attributes and selected characteristics

Variable	Stability	Attachment	Autonomy	Achievement	Enjoyment
Financial worries in the last 12 months	−0.29**	−0.11*	−0.09	−0.14**	−0.15**
Marital status single	−0.30**	−0.28**	−0.10*	−0.15**	−0.18**
Self-care problems	−0.07	−0.11*	−0.37**	−0.20**	−0.18**
No certificated qualifications	0.02	−0.066	−0.11*	−0.15**	−0.11*
Unhappy	−0.48**	−0.41**	−0.20**	−0.34**	−0.45**

* significant at the 5 % level

** significant at the 1 % level

Table 10 reports the differences between the hypothesised strongest correlation and the other correlation coefficients in the row. It can be seen that financial worries correlated more strongly with *stability* than any of the other capabilities. Similarly, self-care is more strongly correlated with *autonomy* than any of the other four capabilities. Education is most strongly correlated with *achievement* as expected, but the differences in correlation are small. In the case of marital status and happiness, *stability*, rather than the hypothesised capability (*attachment* and *enjoyment*, respectively), is marginally more strongly related to the background variable, but there is no evidence that this difference is greater than expected by chance.

**Table 10:** Effect sizes for differences in correlation between hypothesised strongest correlation and other correlations in row

Variable	Stability	Attachment	Autonomy	Achievement	Enjoyment
Financial worries in the last 12 months	–	0.18**	0.21**	0.16*	0.14*
Marital status single	−0.024	–	0.19**	0.13	0.099
Self-care problems	0.32**	0.28**	–	0.19**	0.21**
No certificated qualifications	0.17*	0.087	0.044	–	0.042
Unhappy	−0.038	0.039	0.27**	0.12	–

* significant at the 5 % level

** significant at the 1 % level

## Discussion

This study represents a first investigation of whether capability wellbeing can be captured in a valid manner through a simple generic measure for the (UK) adult population. Although measuring ‘capability’ is challenging [10, 14], the findings indicate that capabilities, self-reported through the ICECAP-A measure, are associated with other indicators of freedom and, in general, socio-economic and health characteristics that were anticipated to be associated with an individual’s capability. The findings therefore provide encouraging evidence of the construct validity of the ICECAP-A measure in this setting.

The correlations between the freedom questions and self-reported capabilities suggest that capability questions appear to ‘capture’ freedom, to a greater degree, than measures of happiness and health do. This may be important when selecting outcome measures in contexts where expanding individuals’ freedoms is a key policy goal. For example, current health policy in England seeks to expand patient choice through the use of personal budgets and involving patients in decisions about the location of their care [29]. It must be noted that the ‘gain’ offered by the capability measure, relative to measures of health and happiness, is, in general, small and (from the *p* values) based on weak statistical levels of evidence. Further investigation is recommended in this area, in particular to employ larger sample sizes and to examine the effect of phrasing questions in terms of capability as opposed to functioning.

A large number of hypotheses were tested, relating to the relationships between individuals’ socio-economic and health characteristics and their responses to capability questions. Although a minority of associations would be expected by chance, 69 % of stated hypotheses (67/97) were found to have *p* values less than 5 %. This provides evidence that the ICECAP-A measure reflects expected differences between individuals in the *general population* defined by their health and education, extending the findings of previous studies of capabilities for older people [16, 17]. Furthermore, this study additionally demonstrated the sensitivity of self-reported capability responses to characteristics such as employment, income, relationships and a range of major life events which provides encouraging evidence for the use of the ICECAP-A as a measure of wellbeing outside (as well as inside) the health setting.

Despite supportive evidence for the majority of hypotheses, a number of anticipated associations were not detected in this sample. Two such areas were between local deprivation [as measured by the domain indices of the IMD (Index of Multiple Deprivation)] and capability, and health care access and capability. One explanation is that the IMD domain indices may be relatively poor proxies for the underlying construct of interest (individual deprivation). On healthcare access, only two of eight hypothesised associations between a recent inpatient/outpatient appointment and capability were detected. One explanation may be that the relationship between health care appointments and wellbeing is complex: although those in ‘need’ of health care may report lower wellbeing than those who do not, those who receive health care are likely to report higher wellbeing than those who do not but have similar ‘need’. Examining the relationship between health care access and wellbeing is confounded by these two relationships.

As this study is the first study to report on the validity of the ICECAP-A measure, there are a number of caveats and research opportunities that are worth noting. First, the method of sampling ensured that individuals had an equal probability of being approached for the survey. However, due to higher response rates in certain groups, older people and females are slightly over-represented in the final sample. Second, other (non-ICECAP) capability measures [11, 30] may exhibit different properties in terms of their sensitivity to characteristics of the population of interest. Furthermore, other capability measures may operate on a different conceptual level, treating health itself as a capability and thus requiring different constructs to be developed to examine validity. Similar assessments of the validity of these alternative measures would be valuable. Third, construct validity, which this study focuses primarily on, is only one measurement property amongst many. Further investigation is required to establish whether capability responses are reliable and whether they are responsive to important changes in an individual’s life over time (for example an episode of poor health or health care intervention). Trial data would be a good vehicle for examining this issue. In general, further work could establish whether self-reported capabilities exhibit desirable validity and acceptability in policy-relevant sub-groups of the population, such as patients, social care recipients and informal carers. Finally it should be noted that this study was conducted through face-to-face interviews. Although the intention is that the ICECAP-A measure would be used in paper-based and internet formats too, further work to assess the validity of the measure in these settings would be useful.

This study does not provide a definitive judgement that capability measurement is valid, since there is substantial debate surrounding the interpretation of capabilities. It does, however, offer a body of evidence suggesting that policy-relevant differences in wellbeing can be identified by the ICECAP-A measure and thus that it offers promise as a tool for capturing outcomes for economic evaluations.

## Appendix 1: ICECAP-A capability wellbeing measure

About your overall quality of life

Please indicate which statements best describe your overall quality of life at the moment by placing a tick (✓) in **ONE** box for each of the five groups below

Reference: [13].

## Abbreviations

ICECAP-A: ICEpop CAPability measure for Adults
